# Morphologic and molecular study on the lens anterior capsule in systemic sclerosis

**DOI:** 10.5935/0004-2749.20210039

**Published:** 2021

**Authors:** Beatriz Fiuza Gomes, Armando S. Crema, Marcony R. Santhiago, Adroaldo Alencar Costa Filho, Haroldo Vieira Moraes Jr, Doralice Silva Paiva, Nádia C.O. Miguel

**Affiliations:** 1 Department of Ophthalmology, Universidade Federal do Rio de Janeiro, Rio de Janeiro, RJ, Brazil; 2 Department of Ophthalmology, Hospital Federal de Bonsucesso, Rio de Janeiro, RJ, Brazil; 3 Oftalmorio, Rio de Janeiro, RJ, Brazil; 4 Department of Ophthalmology, Universidade de São Paulo, São Paulo, SP, Brazil; 5 Pharmacy Faculty, Universidade Federal do Rio de Janeiro, Rio de Janeiro, RJ, Brazil; 6 Institute of Biomedical Sciences, Universidade Federal do Rio de Janeiro, Rio de Janeiro, RJ, Brazil

**Keywords:** lens capsule, crystalline, scleroderma, systemic, Heparinase, heparin lyase, Cápsula do cristalino, Escleroderma sistêmico, Heparinase, Heparina Liase

## Abstract

This study aimed to analyze the anterior lens capsule specimens from both eyes of a patient with systemic sclerosis and compare them to the eyes of a control patient. No significant differences between systemic sclerosis and control eyes were observed in the results from the hematoxylin-eosin and picrosirius staining. In the samples obtained from both systemic sclerosis and control eyes, there were expressions of caspase, a molecule expressed in cell death by apoptosis. Heparanase was overexpressed in the systemic sclerosis sample compared to the control sample. Therefore, the anterior lens capsule of the patient with systemic sclerosis is probably affected by the disease since it showed marked expression of heparanase 1.

## Abstract (pt)

Analisamos as amostras das cápsulas anteriores do cristalino de uma paciente com esclerose sistêmica e com paramos com as de um paciente controle. Não foram observadas diferenças significativas entre esclerose sistêmica e controle nos resultados da coloração com hematoxilina-eosina e picrosirius. Nas amostras obtidas da esclerose sistêmica e do controle, obtivemos expressão de caspase, uma molécula expressa na morte celular por apoptose. A heparinase foi ex pressa de forma mais marcante na amostra de esclerose sistêmica quando comparada ao controle. Portanto, a cápsula anterior do cristalino da paciente com esclerose sistêmica provavelmente foi afetada pela doença, uma vez que mostrou expressão aumentada de heparinase 1.

## INTRODUCTION

Systemic sclerosis (SSc) is a connective tissue disease of unknown origin with heterogeneous manifestations, characterized by immune activation, vasculopathy, and excessive fibrosis^(1)^. Cataract is found in 42%-52% of patients with SSc^(2-5)^. It is not known whether cataract in SSc is related to the disease itself, age, or systemic steroid treatment^(4-6)^. It is also unknown whether the lens capsule of patients with SSc is affected^(4,5)^.

This pilot study aimed to analyze anterior lens capsule specimens from both eyes of a patient with SSc obtained at the time of femtosecond laser-assisted cataract surgery (FLACS) and compare them to those of a control patient matched for age and sex. To the best of our knowledge, the anterior lens capsules of patients with SSc have never been previously studied.

## CASE REPORT

Written informed consent was obtained preoperatively, and the study was conducted in compliance with the Declaration of Helsinki.

This pilot study analyzed both eyes of a 69-year-old woman diagnosed with SSc, who presented to our clinic for FLACS. The patient had been diagnosed with SSc for 5 years and had nuclear cataract on both eyes. As a control, we also examined both eyes of a healthy woman of the same age, who also submitted to FLACS. Biomicroscopically, all eyes showed a comparable degree of opacification in all lens layers.

All surgeries were performed by the same surgeon with the same technique, using the Infinity phacoemulsification machine (Alcon Laboratories) under topical anesthesia. In FLACS, we used the Len-Sx^®^ femtosecond laser (Alcon Laboratories Inc., Fort Worth, TX). A 4.8-mm-diameter capsulotomy procedure was performed. Pulse energy of 6.5 µJ, 5-µm spot separation, and 5-µm layer separation were used for the capsulotomies. The laser parameters were similar in all cases. Surgery was concluded with standard nucleus and cortex removal and implantation of a three-piece hydrophobic acrylic posterior chamber lens in the capsular bag, with complete removal of viscoelastic material by irrigation aspiration. The samples of the lens capsule were stained with hematoxylin-eosin (H&E) for analysis under routine optical microscopy. Picrosirius red (PSR) staining was used to visualize collagen under confocal microscopy. Apoptosis was evaluated by fluorescent staining and microscopy (caspase 3 activity assay). Heparanase 1 expression was also investigated.

### Routine histology

Immediately after surgical removal, the samples were fixed in 4% paraformaldehyde for optical microscopy. The samples were rinsed, dehydrated at high ethanol concentrations, bleached in xylene, and embedded in paraffin. Then, 6.0-mm sections were cut, mounted on gelatin-coated slides, and stained with H&E for analysis under routine optical microscopy. The slides were analyzed by light field microscopy and polarization (Zeiss [AxioScopeA1]).

### PSR staining

For morphological analysis of the sample, the sections were stained with 0.1% PSR (Direct Red 80; Sigma Aldrich). The slides were analyzed by confocal microscopy (Leica TCS SP5). Images were obtained using Fiji software.

### Immunofluorescence analysis

Paraffin sections obtained as described above (routine histology) were dewaxed, cleared in xylene, dehydrated in a graded ethanol series, rinsed with distilled water, and immersed in sodium citrate buffer solution (pH 6.0). Then, the slides were heated in a microwave oven for 5 min at power 10, after which the slides were cooled for 30 min in ammonium chloride solution. Nonspecific antigen blocking was achieved with 5% bovine albumin in phosphate buffered saline (PBS)/triton for 60 min. Subsequently, the primary antibodies, caspase-3 (Millipore) and heparanase 1 (Santa Cruz Biotechnology), were added and left in a wet chamber at approximately 4°C overnight.

On the next day, after three washes with PBS, the secondary antibody was added for 2 h (anti-rabbit Alexa Fluor 488). Four consecutive washes with PBS were performed; then, a solution of 2-(4-amidinophenyl)1H-in dole-6-carboxamidine (DAPI) was placed to mark the cell nuclei. The slides were mounted using Fluoromount-G™ (Southern Biotech, Birmingham, AL, USA) and examined under a confocal microscope (Leica TCS SPE, Leica Microsystems, Bannockburn, IL, USA). The images were obtained using Fiji software.

## RESULTS

**Figure 1:**
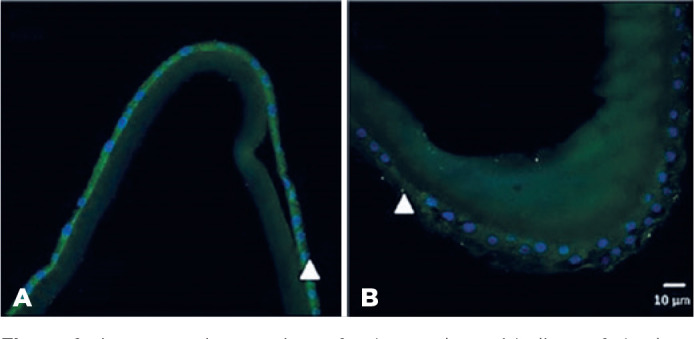
A panoramic overview of subcapsular epithelium of the lens capsule with hematoxylin-eosin (H&E) staining in a patients with systemic sclerosis (SSc) (A) and control patient (B) and the results for picrosirius staining observed in confocal microscopy in a patient with SSc (C) and control patient (D). No significant differences between patients with systemic sclerosis and controls were observed in the results of H&E and picrosirius staining.

**Figure 2:**
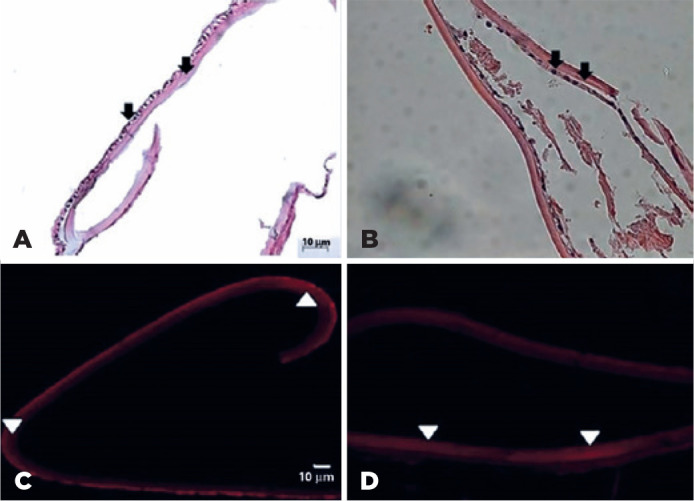
Results of immunofluorescence analysis. In the systemic sclerosis (SSc) (A) and control (B) samples, there were expressions of caspase, a molecule expressed in cell death by apoptosis. Heparanase was overexpressed in the SSc sample (C) compared to control sample (D).

No capsule structural abnormalities were observed in the samples of the patient with SSc on optical microscopy. Figure 1 shows a panoramic overview of subcapsular epithelium of lens capsule with H&E staining (1) and the results of PSR staining (2) observed in confocal in SSc patient (A) and control patient (B). No meaningful differences between the SSc and control samples were observed in the results of H&E and PSR staining. The amount of collagen in the lens capsule seems to be the similar in the patient with SSc and control. Figure 2 shows the results of immunofluorescence analysis. In all samples, there were expressions of caspase, a molecule expressed in cell death by apoptosis. Heparanase 1 expression was markedly more important in SSc than in the control.

## DISCUSSION

Lens opacity in SSc mostly develop in the form of posterior subcapsular cataract and nuclear cataract, while cortical cataract appearing as focal cystic opacities develops less frequently^(5)^. A study has shown that posterior subcapsular cataract is significantly more common in patients with SSc compared to the control group^(5)^. A higher incidence of this cataract form in patients with SSc is believed to result from lens nutrition disorders secondary to narrowing of ciliary body vessels and/or complications of general steroid treatment applied in such cases^(5)^.

The connective tissue changes that occur in SSc may also affect the lens capsule. The human lens capsule is a base membrane composed mainly of type IV collagen that is secreted by lens epithelial cells, which exist in a single layer^(7)^. Epithelial cells in the capsule may indicate a greater change in opacity^(7)^.

In this study, besides H&E staining, PSR staining was used to demonstrate the collagen fibers in the lens capsule. No significant differences were found in the morphological analysis of the lens capsule between the SSc and control samples.

Studies have demonstrated that the human lens epithelium undergoes cell death after anterior lens capsule extraction during FLACS^(8)^. This is in accordance with our results that showed expressions of caspase, a molecule expressed in cell death by apoptosis in the SSc and control samples, probably related to FLACS.

Because heparanase-mediated biological processes seemed to be involved in the development of SSc^(9)^, we investigated the expression of heparanase in the anterior lens capsule. Heparanase is an endo-β-D-glucuronidase that cleaves specific linkages in the structure of heparin sulfate (HS), one of the extracellular matrix (ECM) components, yielding fragments that are able to contain biological activity. HS cleavage results in remodeling of the ECM and regulating the release of many HS-linked molecules, such as growth factors, cytokines, and enzymes involved in inflammation, wound healing, and tumor invasion^(10)^. With a few exceptions, in normal noncancerous cells, the heparanase gene is not transcribed. Heparanase expression is induced in all major types of human cancer and has been associated with numerous inflammatory conditions, such as inflammatory bowel disease and rheumatoid arthritis^(9)^. With respect to its expression in ocular tissues, it is of note that corneas of patients with keratoconus significantly overexpressed heparanase at both the epithelial and stromal levels. Of note, a different study presented at ASCRS meeting reported a higher prevalence of keratoconus in patients with SSc compared to controls^(11)^.

The precise mode of action of heparanase upregulation is unclear^(10)^. In patients with SSc, serum heparanase levels are also significantly higher than in healthy individuals^(12)^. Heparanase has been shown to be involved in the regulation of angiogenesis under the physiological and pathological conditions. It primarily promotes the release of ECM-resident angiogenic factors, including basic fibroblast growth factor and VEGF. In this way, heparanase potentially contributes to the development of SSc^(9)^.

Therefore, heparanase overexpression was found in the anterior lens capsule of both eyes of a patient with SSc.
